# The impact of obesity on the relationship between epicardial adipose tissue, left ventricular mass and coronary microvascular function

**DOI:** 10.1007/s00259-015-3087-5

**Published:** 2015-06-09

**Authors:** M. J. Bakkum, I. Danad, M. A. J. Romijn, W. J. A. Stuijfzand, R. M. Leonora, I. I. Tulevski, G. A. Somsen, A. A. Lammertsma, C. van Kuijk, A. C. van Rossum, P. G. Raijmakers, P. Knaapen

**Affiliations:** Department of Cardiology, VU University Medical Center, Amsterdam, The Netherlands; Cardiology Centers of the Netherlands, Amsterdam, The Netherlands; Department of Radiology & Nuclear Medicine, VU University Medical Center, Amsterdam, The Netherlands

**Keywords:** Coronary microvascular function, Epicardial adipose tissue, Left ventricular mass, Obesity, [^15^O]H_2_O Positron emission tomography

## Abstract

**Purpose:**

Epicardial adipose tissue (EAT) has been linked to coronary artery disease (CAD) and coronary microvascular dysfunction. However, its injurious effect may also impact the underlying myocardium. This study aimed to determine the impact of obesity on the quantitative relationship between left ventricular mass (LVM), EAT and coronary microvascular function.

**Methods:**

A total of 208 (94 men, 45 %) patients evaluated for CAD but free of coronary obstructions underwent quantitative [^15^O]H_2_O hybrid positron emission tomography (PET)/CT imaging. Coronary microvascular resistance (CMVR) was calculated as the ratio of mean arterial pressure to hyperaemic myocardial blood flow.

**Results:**

Obese patients [body mass index (BMI) > 25, *n* = 133, 64 % of total] had more EAT (125.3 ± 47.6 vs 93.5 ± 42.1 cc, *p* < 0.001), a higher LVM (130.1 ± 30.4 vs 114.2 ± 29.3 g, *p* < 0.001) and an increased CMVR (26.6 ± 9.1 vs 22.3 ± 8.6 mmHg×ml^−1^×min^−1^×g^−1^, *p* < 0.01) as compared to nonobese patients. Male gender (β = 40.7, *p* < 0.001), BMI (β = 1.61, *p* < 0.001), smoking (β = 6.29, *p* = 0.03) and EAT volume (β = 0.10, *p* < 0.01) were identified as independent predictors of LVM. When grouped according to BMI status, EAT was only independently associated with LVM in nonobese patients. LVM, hypercholesterolaemia and coronary artery calcium score were independent predictors of CMVR.

**Conclusion:**

EAT volume is associated with LVM independently of BMI and might therefore be a better predictor of cardiovascular risk than BMI. However, EAT volume was not related to coronary microvascular function after adjustments for LVM and traditional risk factors.

**Electronic supplementary material:**

The online version of this article (doi:10.1007/s00259-015-3087-5) contains supplementary material, which is available to authorized users.

## Introduction

Left ventricular mass (LVM) has been shown to be a strong predictor of adverse cardiovascular outcomes and has been demonstrated to remain a potent prognosticator even after the adjustment for hypertension and other traditional risk factors [1]. There is a large body of literature documenting an increase in LVM in obese individuals [2, 3]. While the aetiology of this link between obesity and LVM remains elusive, the visceral fat between the myocardium and visceral pericardium known as epicardial adipose tissue (EAT) may be a potential contributing factor. Like visceral abdominal fat, EAT appears to be increased in obesity [4, 5]. It has previously been demonstrated that EAT plays a role in coronary atherosclerosis through a paracrine manner, by the secretion of pro-inflammatory cytokines [6]. These local effects, however, may not be limited to the coronary vasculature and may also influence the underlying myocardium [7]. Indeed, there is some evidence that EAT contributes to LV remodelling and Iacobellis et al. showed a relationship between echocardiographically measured EAT and LVM in healthy volunteers [8]. Furthermore, EAT was also found to be predictive of an impaired coronary vasodilator capacity in patients without obstructive coronary artery disease (CAD) [9, 10]; hence, a key role of EAT in the development of coronary microvascular dysfunction has been postulated. However, persuasive studies on the quantitative relationship between EAT, LVM and coronary microvascular function are lacking. Therefore, the present study aimed to determine the relationship between EAT, LVM and coronary microvascular function, as measured by hybrid [^15^O]H_2_O positron emission tomography (PET)/CT imaging, in patients evaluated for CAD in whom haemodynamically significant disease was excluded.

## Materials and methods

### Patient population

**Fig. 1:**
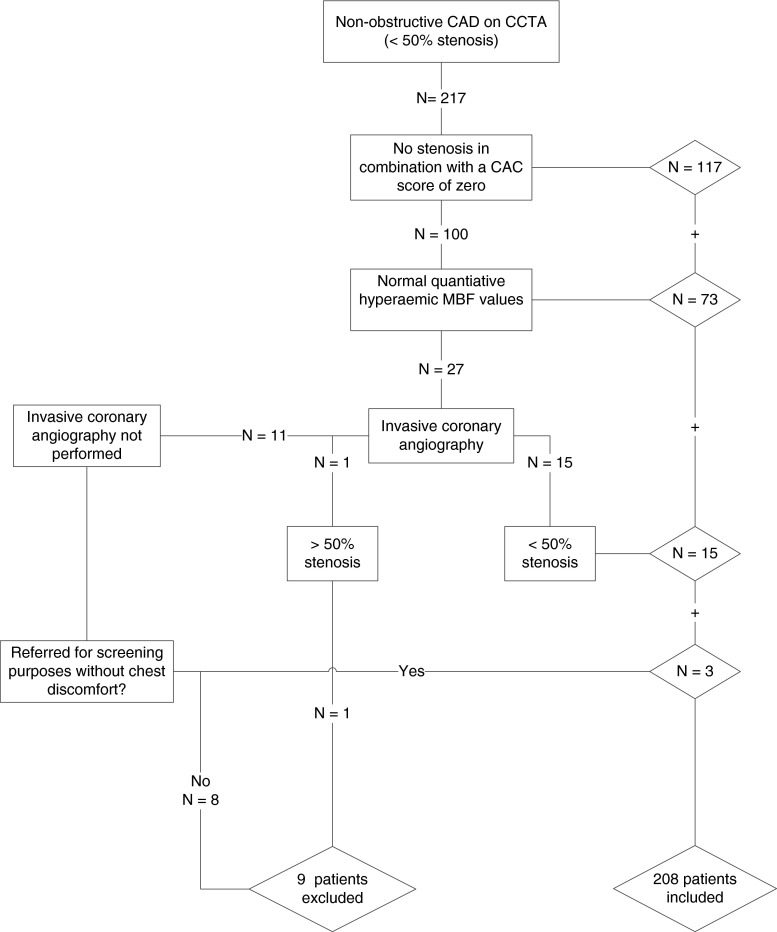
Flow diagram of patient inclusion. Steps taken to exclude any obstructive CAD among patients. Patients were included if, in addition to a negative CCTA scan, they exhibited: (1) no calcifications or (2) a normal hyperaemic MBF or (3) a negative invasive coronary angiography or (4) no symptoms. A total of 208 patients were included in the study. *CAD* coronary artery disease, *CCTA* coronary computed tomography angiography, *CAC* coronary artery calcium, *MBF* myocardial blood flow

Data were retrospectively obtained from a clinical cohort of patients being referred for cardiac hybrid [^15^O]H_2_O PET/CT imaging because of symptoms suggestive of angina pectoris or a high risk profile (i.e. presence of two or more risk factors in the absence of symptoms). Hypertension was defined as a blood pressure of ≥ 140/90 mmHg or the use of antihypertensive medication. Hypercholesterolaemia was defined as a total cholesterol level of ≥ 5 mmol/l or treatment with cholesterol-lowering medication. Patients were classified as having diabetes if they were receiving treatment with oral hypoglycaemic drugs or insulin. A history of smoking was allocated to all current and former smokers. A positive family history was defined as having at least one first-degree relative with CAD before the age of 55 in a male or below the age of 65 in a female relative. Obesity was defined as a body mass index (BMI) ≥ 25. Patients with cardiomyopathy, impaired LV function and/or a documented history of CAD were excluded. A history of CAD was defined as a previous myocardial infarction, percutaneous coronary intervention or coronary artery bypass graft surgery. Furthermore, patients with obstructive CAD on coronary computed tomography angiography (CCTA) were excluded. Obstructive CAD on CCTA was considered to have been ruled out on a CT scan of sufficient quality enabling adequate grading of all major coronary segments, which did not display a stenosis ≥ 50 %. A total of 217 patients met this criterion. To ensure the exclusion of myocardial ischaemia, patients were only eligible for the study if they had no coronary calcifications (coronary calcium score = 0) and absence of non-calcified coronary plaques [11]. If not, only patients with hyperaemic myocardial blood flow (MBF) values > 2.3 ml×min^−1^×g^−1^ were included. In the presence of abnormal myocardial MBF, non-obstructive CAD (<50 % stenosis) on the invasive coronary angiogram was considered to adequately rule out functionally relevant epicardial disease. Finally, patients with evidence of coronary atherosclerosis, but who were referred for screening purposes only and who had no chest discomfort, were also included in the study. In 9 of the initial 217 patients haemodynamically significant CAD could not be excluded with certainty and they were therefore excluded from the analysis. As such, a total of 208 patients met the inclusion criteria and are described in the present study. A flowchart of the inclusion process is demonstrated in Fig. 1. The pretest likelihood of CAD was determined using the Diamond and Forrester score, using <13.4 % , 13.4–87.2 % and > 87.2 % as cut-off values for low, intermediate and high likelihood for CAD, respectively [12]. The requirement for informed consent was waived by the Ethics Committee due to the retrospective nature of the study.

### Scan protocol

**Fig. 2:**
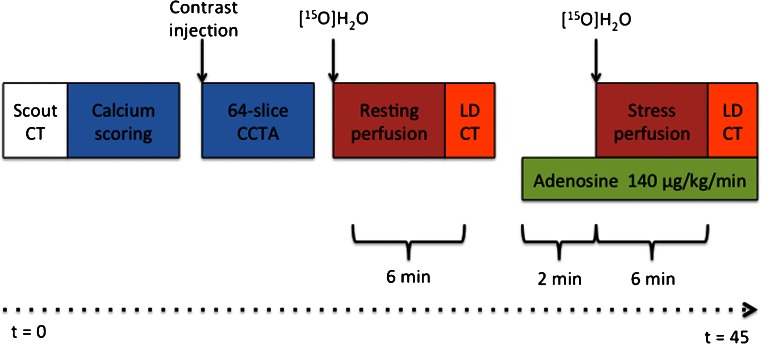
Hybrid PET/CCTA protocol. After a scout CT for patient positioning a non-contrast (calcium scoring) and contrast-enhanced CT scans were sequentially performed. This was followed by a [^15^O]H_2_O PET myocardial perfusion scan in resting conditions and a low-dose CT scan for attenuation correction. A minimum of 10 min after the first dose of [^15^O]H_2_O, to allow for radiation decay, an identical PET sequence was commenced for hyperaemic perfusion. Adenosine infusion at 140 μg×kg^−1^×min^−1^ was started 2 min before the start of the dynamic PET sequence

All patients underwent a PET/CT scanning protocol for the concomitant evaluation of coronary anatomy and myocardial perfusion. All patients were instructed to refrain from caffeine and xanthine derivates 24 h prior to the scans. The hybrid PET/CT imaging protocol is shown in Fig. 2 and has been described in detail previously [13, 14].

### Cardiac CT

**Fig. 3:**
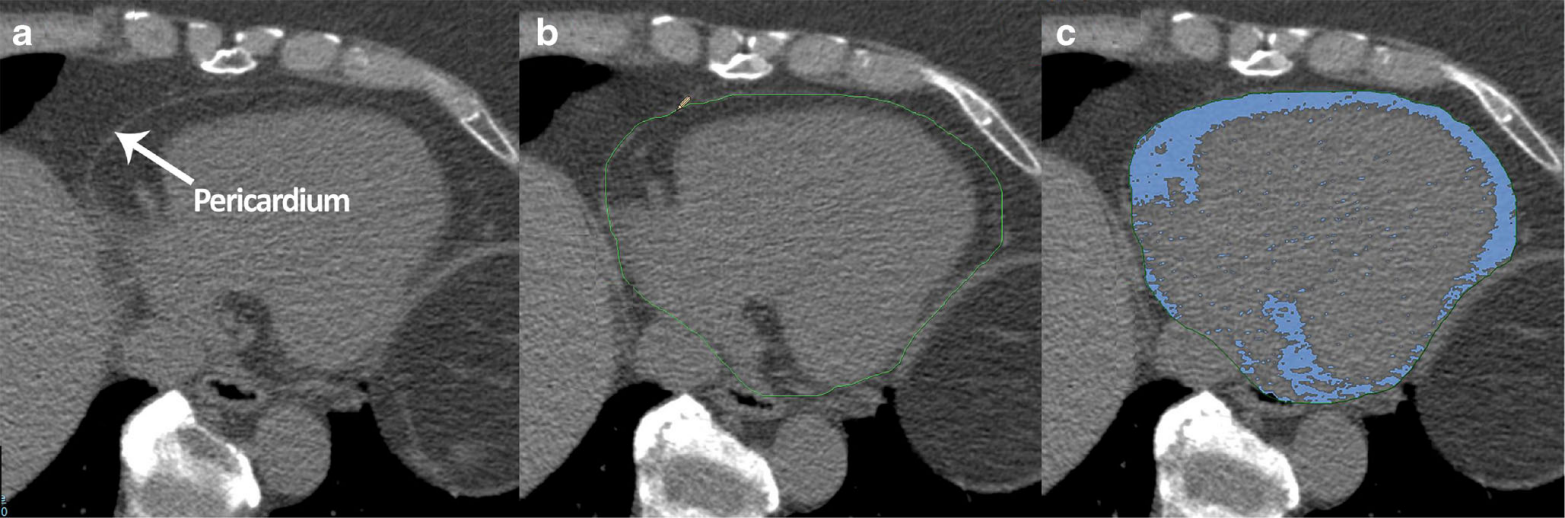
Example of EAT quantification in one axial slice. The pericardium was identified (**a**) and traced manually (**b**). The adipose tissue within the region of interest (indicated in *blue*) was then automatically quantified and multiplied by the slice thickness (2.5 mm) (**c**). Summing the EAT of all slices between the pulmonary trunk and lowest slice showing the posterior descending artery gave the total EAT volume. All measurements were performed using Phillips IntelliSpace workstation v5.0 (Philips Healthcare, Best, The Netherlands)

After a scout CT for patient positioning all patients underwent a non-contrast CT scan for coronary artery calcium (CAC) scoring followed by a CCTA scan. A bolus of 100 ml iodinated contrast agent (Xenetix 350, Guerbet, Paris, France) was injected intravenously at an injection rate of 5 ml×s^−1^ followed by a 50-ml saline chaser. A standard scanning protocol was applied, with 64 × 0.625 mm section collimation, 420-ms gantry rotation time, 120-kV tube voltage and a tube current of 800–1,000 mA, depending on the patient’s body size. The automatic bolus triggering technique was used to initiate image acquisition. To reduce radiation dose, an ECG-gated tube current modulation was used. EAT was measured on the non-contrast CT images using dedicated volumetric software (Philips IntelliSpace workstation v5.0, Philips Healthcare, Best, The Netherlands). The most cranial slice was at the level of the pulmonary trunk bifurcation, and the most caudal slice was identified as the last slice in which the posterior descending artery was visible. The method for quantifying EAT volume has been described in detail previously [15]. An example of EAT quantification is shown in Fig. 3. In addition, LVM was automatically segmented from the CCTA images. All analyses were performed on a Philips IntelliSpace workstation v5.0 (Philips Healthcare, Best, The Netherlands).

### PET imaging

The CCTA protocol was followed by PET myocardial perfusion imaging (Fig. 2). After the injection of 370 MBq of 5 ml [^15^O]H_2_O at a rate of 0.8 ml×s^−1^ bolus, which was immediately followed by a 35-ml saline flush (2 ml×s^−1^), a 6-min emission scan was started during resting conditions. This dynamic scan sequence was followed immediately by a respiration-averaged slow low-dose CT scan to correct for attenuation. After an interval of 10 min to allow for decay of radioactivity, adenosine was administered intravenously to induce hyperaemia. Two minutes after the start of intravenous adenosine infusion of 140 μg×kg^−1^×min^−1^, a dynamic 6-min PET sequence was started. All images were reconstructed using the 3-D row action maximum likelihood algorithm into 22 frames (1 × 10, 8 × 5, 4 × 10, 2 × 15, 3 × 20, 2 × 30 and 2 × 60 s), applying all appropriate corrections. Parametric MBF images were generated and quantitative data were generated using in-house developed software, namely Cardiac *VU*er [16]. MBF was expressed in ml×min^−1^×g^−1^ of perfusable tissue for each of the three vascular territories and for the entire left ventricle.

### Image interpretation

All CT scans were analysed by an experienced radiologist and cardiologist. The axial slices were initially evaluated for the presence of significant segmental disease and, additionally, multiplanar and curved multiplanar reconstructed images were used to determine stenosis severity. The coronary tree was evaluated according to a 16-segment coronary artery model modified from the American Heart Association [17]. CT-defined obstructive CAD was excluded if the segments contained no CAD (no stenosis in combination with a CAC score of zero) or non-obstructive CAD, which is defined as a coronary stenosis causing a luminal diameter reduction < 50 %. Two experienced readers visually graded the parametric PET stress perfusion images for the presence of a perfusion defect resembling myocardial ischaemia, according to the 17-segment model of the American Heart Association [18]. After visual assessment of the images, the readers interpreted the quantitative perfusion data. The quantitative analysis allows for the measurement of MBF for the calculation of coronary flow reserve (CFR) and hyperaemic coronary microvascular resistance (CMVR). The CFR is defined as the ratio of hyperaemic MBF to baseline perfusion, whereas CMVR was determined by dividing mean arterial pressure by hyperaemic MBF [19]. For the exclusion of myocardial ischaemia due to flow-limiting epicardial lesions, a hyperaemic MBF value < 2.3 ml×min^−1^×g^−1^ was considered abnormal. This cut-off value has previously been determined against a background of fractional flow reserve [20]. These quantitative results were combined with the visual grading of PET images and in conjunction with the coronary anatomy as obtained with CCTA to obtain a hybrid interpretation.

### Statistical analysis

All continuous variables are reported as mean values ± standard deviation (SD) and categorical variables are presented as their actual value and percentage of group total. Evaluation of relationships between variables was performed using Student’s *t* tests, chi-square tests and Pearson’s correlation analyses. For reproducibility analyses, inter- and intraobserver correlations were assessed using intraclass correlation coefficients in 53 randomly selected patients. Linear regression models for outcome LVM were constructed, including the following parameters in the univariable and multivariable analyses: traditional risk factors (including age, gender, BMI, hypercholesterolaemia, hypertension, smoking, family history and diabetes), CAC and EAT. Linear regression analyses for outcome CMVR were performed with traditional risk factors, CAC, EAT, and LVM. For heterogeneity estimation, analysis of variance (ANOVA) was used to compare hyperaemic MBF per coronary artery. Additionally, coefficients of variation (COV) were calculated by dividing the SD of hyperaemic MBF by the mean hyperaemic MBF on a per-coronary basis. A two-sided *p* value of <0.05 was considered significant. Statistical analyses were performed with IBM SPSS Statistics version 20 (IBM SPSS Statistics, IBM Corporation, Armonk, NY, USA).

## Results

### Baseline characteristics

Baseline patient data are listed in Table 1 (demographics) and Table 2 (imaging characteristics). A total of 208 patients are described in the current study of whom 133 (64 %) had a BMI ≥ 25. Obese patients had a higher LVM (130.1 ± 30.4 vs 114.2 ± 29.3, *p* < 0.001), more EAT (125.3 ± 47.6 vs 93.5 ± 42.1, *p* < 0.001), a lower hyperaemic MBF (3.23 ± 1.42 vs 3.80 ± 1.42, *p* < 0.01) and a higher CMVR (26.6 ± 9.1 vs 22.3 ± 8.6, *p* < 0.01) as compared to the nonobese patients.

**Table 1:** Baseline patient characteristics

	All patients	Lean patients (BMI < 25)	Overweight patients (BMI ≥25)	*p* value (between obese and nonobese patients)
	*n* = 208	*n* = 75	*n* = 133	
Male gender	94 (45 %)	30 (40 %)	64 (48 %)	0.31
Age	55.1 ± 9.4	54.4 ± 9.6	55.4 ± 9.3	0.44
BMI	26.5 ± 4.1	22.7 ± 1.7	28.7 ± 3.4	<0.001
Pretest likelihood for CAD	43.1 ± 29.7	39.6 ± 28.9	45.1 ± 30.1	0.20
Low	33 (16 %)	15 (20 %)	18 (14 %)	0.24
Intermediate	157 (76 %)	55 (73 %)	102 (77 %)	0.62
High	18 (9 %)	5 (7 %)	13 (10 %)	0.61
Risk factors				
Diabetes	32 (15 %)	3 (4 %)	29 (22 %)	<0.001
Hypertension	69 (33 %)	15 (22 %)	54 (41 %)	<0.01
Hypercholesterolaemia	60 (29 %)	13 (18 %)	47 (35 %)	<0.01
Smoking	83 (40 %)	32 (43 %)	51 (38 %)	0.55
Family history of CAD	109 (52 %)	34 (46 %)	75 (57 %)	0.15
Reason for referral				
Typical AP	56 (27 %)	18 (24 %)	38 (29 %)	0.52
Atypical AP	65 (31 %)	22 (29 %)	43 (32 %)	0.76
Aspecific AP	67 (32 %)	32 (43 %)	35 (26 %)	0.02
Screening/high-risk profile	20 (10 %)	3 (4 %)	17 (13 %)	0.05

*BMI* body mass index, *CAD* coronary artery disease, *AP* angina pectoris

**Table 2:** Baseline quantitative [^15^O]H_2_O PET/CT imaging results

	All patients	Lean patients (BMI < 25)	Overweight patients (BMI ≥25)	*p* value (between obese and nonobese patients)
	*n* = 208	*n* = 75	*n* = 133	
CT results				
LVM	124.4 ± 30.9	114.2 ± 29.3	130.1 ± 30.4	<0.001
EAT volume	113.8 ± 48.1	93.5 ± 42.1	125.3 ± 47.6	<0.001
CAC score	71.9 ± 290.5	74.2 ± 355.6	70.6 ± 248.1	0.93
No calcifications				
Myocardial perfusion				
Resting MBF	1.07 ± 0.46	1.11 ± 0.4	1.05 ± 0.52	0.37
Hyperaemic MBF	3.44 ± 1.28	3.80 ± 1.42	3.23 ± 1.15	<0.01
CFR	3.44 ± 1.29	3.60 ± 1.36	3.34 ± 1.25	0.18
CMVR	25.1 ± 9.13	22.3 ± 8.60	26.6 ± 9.10	0.001

*LVM *left ventricular mass, *EAT* epicardial adipose tissue, *CAC* coronary artery calcium, *MBF* myocardial blood flow, *CFR* coronary flow reserve, *CMVR* coronary microvascular resistance

### Homogeneity of perfusion

**Fig. 4:**
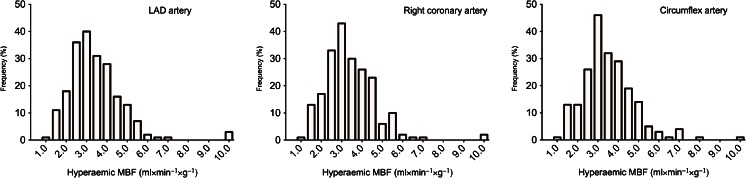
Distribution of hyperaemic MBF. Histograms on the distribution of hyperaemic MBF for the left anterior descending (*LAD*) artery, the right coronary artery and the circumflex coronary artery

The mean hyperaemic MBF did not differ per coronary artery (*p* = 0.64) and was 3.44 ± 1.34 (COV = 38.9 %), 3.39 ± 1.27 (COV = 37.6 %) and 3.51 ± 1.28 (COV = 36.4 %) for the left anterior descending artery, right coronary artery and circumflex coronary artery, respectively (Table 3). The distribution of hyperaemic MBF per coronary is shown in Fig. 4.

**Table 3:** Homogeneity of perfusion patterns

	LAD artery	Right coronary artery	Circumflex artery	*p* value (coronary arteries)
Mean ± SD	3.44 ± 1.34	3.39 ± 1.27	3.51 ± 1.28	0.64
COV	38.9 %	37.6 %	36.4 %	

*SD* standard deviation, *LAD* left anterior descending, *COV* coefficient of variation

### Determinants of LVM

**Fig. 5:**
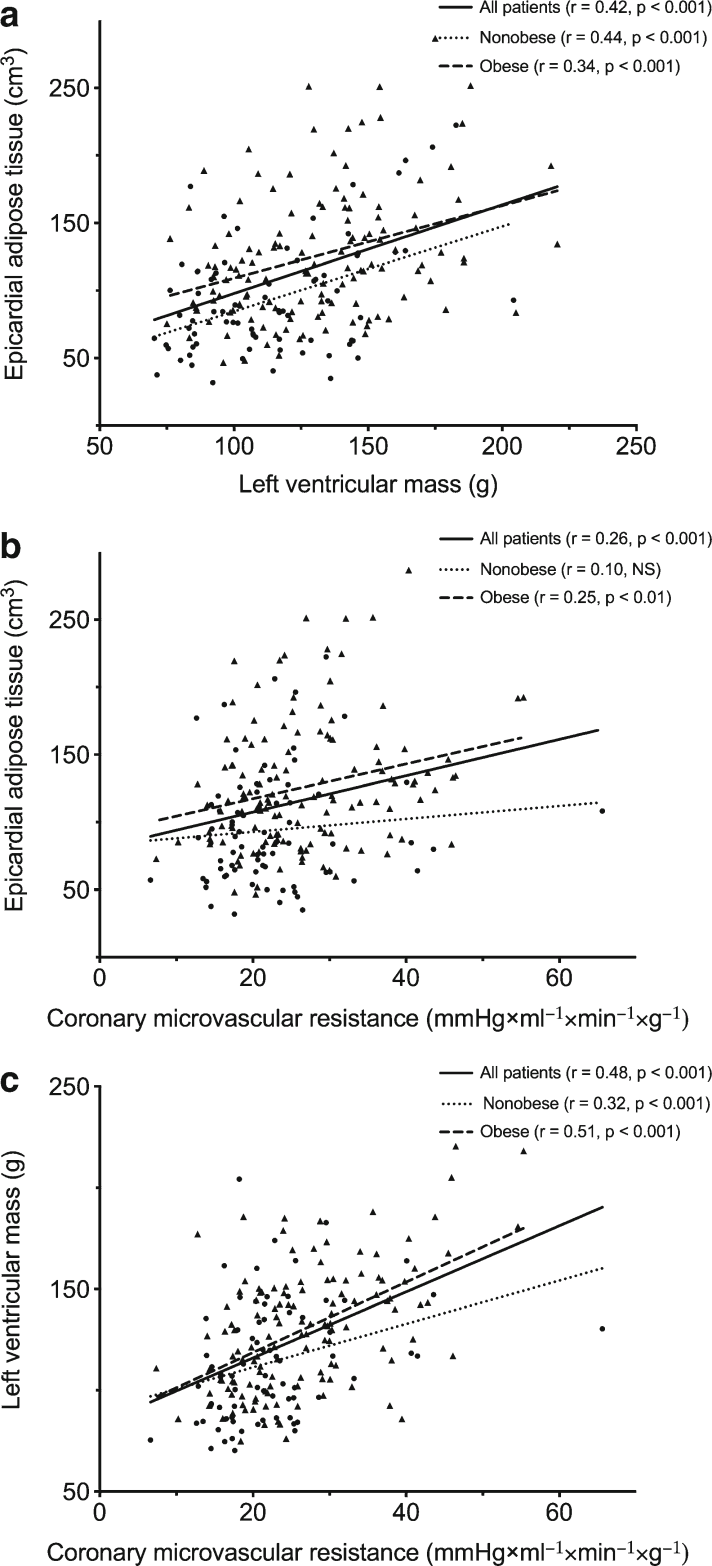
Correlations between EAT, LVM and CMVR for obese and nonobese patients. **a** The relation between EAT and LVM. **b** The relation between EAT and CMVR. **c** The relation between LVM correlated and CMVR. *EAT* epicardial adipose tissue, *LVM* left ventricular mass, *CMVR* coronary microvascular resistance

A significantly positive correlation between EAT and LVM was observed for obese (*R* = 0.44, *p* < 0.001) and nonobese (*R* = 0.34, *p* < 0.001) patients (Fig. 5). Results of univariable linear regression analysis are listed in Table 4. After adjustment traditional risk factors and the CAC score, multivariable regression analysis (*R*^2^ = 0.61) showed male gender (β = 40.7, *p* < 0.001), BMI (β = 1.61, *p* < 0.001), smoking (β = 6.29, *p* = 0.03) and EAT (β = 0.10, *p* < 0.01) to be independent predictors of LVM (Table 4). Of note, when performing the multivariable regression analysis according to BMI groups, EAT was only independently associated with LVM in nonobese patients (β = 0.23, *p* < 0.001), whereas in obese patients male gender (β = 44.2, *p* < 0.001) and BMI (β = 2.03, *p* < 0.001) were predictive of LVM. For both male and female patients, BMI and EAT were identified as independent predictors of LVM, while smoking was identified as a predictor of LVM in female patients only (Supplementary Table 1).

**Table 4:** Univariable and multivariable regression analysis describing the relationship between traditional cardiac risk factors, EAT and LVM

	Univariable analysis			Multivariable analysis		
	β	95 % CI	*p* value	β	95 % CI	*p* value
All patients (*R* ^2^ = 0.61)						
Male gender	44.3	38.3 to 50.2	<0.001	40.7	35.2 to 46.3	<0.001
Age	0.29	−0.74 to 0.17	0.21			NS
BMI	2.74	1.77 to 3.71	<0.001	1.61	0.85 to 2.37	<0.001
Diabetes	12.4	0.77 to 24.1	0.04			NS
Hypertension	7.20	−1.78 to 16.2	0.12			NS
Hypercholesterolaemia	1.47	−7.92 to 10.9	0.76			NS
Smoking	5.60	−3.06 to 14.3	0.20	6.29	0.79 to 11.8	0.03
Family history of CAD	5.10	−13.6 to 3.44	0.24			NS
EAT	0.27	0.19 to 0.35	<0.001	0.10	0.04 to 0.17	<0.01
CAC	0.01	−0.01 to 0.02	0.42			NS
Nonobese patients (*R* ^2^ = 0.65)						
Male gender	40.2	30.0 to 50.4	<0.001	30.9	22.0 to 39.8	<0.001
Age	0.58	−1.23 to 0.12	0.11	−0.54	−0.98 to 0.10	0.02
BMI	5.56	1.79 to 9.33	<0.01			NS
Diabetes	32.4	−66.4 to 1.62	0.06			NS
Hypertension	1.08	−16.0 to 18.2	0.90			NS
Hypercholesterolaemia	1.41	−19.5 to 16.7	0.88			NS
Smoking	21.1	8.1 to 34.1	<0.01	13.4	4.84 to 21.9	<0.01
Family history of CAD	9.76	−23.4 to 3.85	0.16			NS
EAT	0.30	0.16 to 0.45	<0.001	0.23	0.12 to 0.34	<0.001
CAC	0.00	−0.02 to 0.02	0.72			NS
Obese patients (*R* ^2^ = 0.59)						
Male gender	45.0	38.0 to 52.1	< 0.001	44.2	37.4 to 51.0	< 0.001
Age	0.19	−0.75 to 0.38	0.52			NS
BMI	2.43	0.94 to 3.91	< 0.01	2.03	1.04 to 3.03	< 0.001
Diabetes	13.0	052 to 25.5	0.04			NS
Hypertension	5.15	−5.51 to 15.8	0.34			NS
Hypercholesterolaemia	1.86	−12.84 to 9.12	0.74			NS
Smoking	2.19	−13.0 to 8.61	0.69			NS
Family history of CAD	5.10	−15.7 to 5.52	0.34			NS
EAT	0.22	0.12 to 0.33	< 0.001			NS
CAC	0.01	−0.01 to 0.03	0.39			NS

*CI* confidence interval, *BMI* body mass index, *CAD* coronary artery disease, *EAT* epicardial adipose tissue, *LVM* left ventricular mass, *CAC* coronary artery calcium, *NS* not significant

### Determinants of CMVR

There was a significant correlation between CMVR and EAT in obese patients only (*R* = 0.25, *p* < 0.001). A significant increase in CMVR was observed with rising LVM in both obese (*R* = 0.48, *p* < 0.001) and nonobese patients (*R* = 0.32, *p* < 0.01, Fig. 5). Table 5 lists the univariable linear regression analysis for CMVR. Multivariable linear regression analysis revealed that hypercholesterolaemia (β = 2.52, *p* = 0.04), EAT (β = 0.14, *p* < 0.001) and CAC (β = 0.01, *p* < 0.01) are predictive of CMVR. When grouped according to BMI status, these same risk factors remained significantly associated with CMVR in the group of obese patients (Table 5). In the group of nonobese patients, male gender (β = 5.09, *p* < 0.01) and smoking (β = 5.54, *p* < 0.01) were independently associated with CMVR. When, instead of CMVR, CFR was used as marker for coronary function only age (β = −0.03, *p* < 0.01) was identified an as independent predictor for all patients (*R*^2^ = 0.06). When split according to obesity status, age (β = −0.03, *p* < 0.01) remained the only parameter independently associated with CFR for the group of obese patients (*R*^2^ = 0.05), whereas in the group of nonobese patients no risk factors were found to be independently associated.

**Table 5:** Univariable and multivariable regression analysis describing the relationship between traditional cardiac risk factors, EAT, LVM and coronary microvascular function

	Univariable analysis			Multivariable analysis		
	β	95 % CI	*p* value	β	95 % CI	*p* value
All patients (*R* ^2^ = 0.27)						
Male gender	0.72	4.88 to 9.54	< 0.001			NS
Age	0.10	−0.04 to 0.23	0.17			NS
BMI	0.59	0.29 to 0.90	< 0.001			NS
Diabetes	4.61	1.19 to 8.03	< 0.01			NS
Hypertension	3.41	0.77 to 6.06	0.01			NS
Hypercholesterolaemia	3.17	0.39 to 5.94	0.03	2.52	0.10 to 4.93	0.04
Smoking	1.44	−1.15 to 4.02	0.27			NS
Family history of CAD	0.86	−3.41 to 1.69	0.51			NS
EAT	0.05	0.02 to 0.07	< 0.001			NS
LVM	0.14	0.10 to 0.18	< 0.001	0.14	0.10 to 0.17	< 0.001
CAC	0.01	0.00 to 0.01	< 0.01	0.01	0.00 to 0.01	< 0.01
Nonobese patients (*R* ^2^ = 0.19)						
Male gender	5.65	1.76 to 9.54	< 0.01	5.09	1.29 to 8.89	< 0.01
Age	0.01	−0.21 to 0.22	0.97			NS
BMI	0.01	−1.16 to 1.19	0.98			NS
Diabetes	1.09	−11.3 to 9.15	0.83			NS
Hypertension	4.20	−0.73 to 9.13	0.09			NS
Hypercholesterolaemia	0.27	−5.58 to 5.04	0.92			NS
Smoking	6.00	2.16 to 9.85	< 0.01	5.54	1.80 to 9.28	< 0.01
Family history of CAD	1.23	−5.29 to 2.84	0.55			NS
EAT	0.02	−0.03 to 0.07	0.41			NS
LVM	0.09	0.03 to 0.16	< 0.01			NS
CAC	0.00	−0.00 to 0.01	0.17			NS
Obese patients (*R* ^2^ = 0.32)						
Male gender	7.54	4.66 to 10.4	< 0.001			NS
Age	0.13	−0.04 to 0.30	0.13			NS
BMI	0.53	0.07 to 0.99	0.03			NS
Diabetes	4.05	0.32 to 7.79	0.03			NS
Hypertension	2.04	−1.16 to 5.24	0.21			NS
Hypercholesterolaemia	3.43	0.15 to 6.70	0.04	2.94	0.12 to 5.75	0.04
Smoking	0.85	−4.11 to 2.42	0.61			NS
Family history of CAD	1.36	−4.56 to 1.84	0.40			NS
EAT	0.05	0.02 to 0.08	< 0.01			NS
LVM	0.15	0.11 to 0.20	< 0.001	0.15	0.10 to 0.19	< 0.001
CAC	0.01	0.00 to 0.02	< 0.01	0.01	0.00 to 0.01	<0.01

*CI* confidence interval, *BMI* body mass index, *CAD* coronary artery disease, *EAT* epicardial adipose tissue, *LVM* left ventricular mass, *CAC* coronary artery calcium, *NS* not significant

### Reproducibility of EAT measurements

The intraclass correlation coefficient of EAT volume measurements assessed in 53 randomly selected patients were 0.98 (*p* < 0.001) and 0.94 (*p* < 0.001) for intra- and interobserver variability, respectively.

## Discussion

This study evaluated the relationship between EAT, LVM and CMVR in symptomatic patients without obstructive CAD. The main findings of this study are (1) a greater cardiac adipose volume is independently associated with an increase in LVM, (2) EAT is not predictive of coronary microvascular function in symptomatic subjects without obstructive CAD and (3) LVM is associated with coronary microvascular function independently of traditional risk factors and EAT volume.

This study shows that an increase in EAT, the visceral fat depot surrounding the heart, is significantly related to an increase in LVM. This is in accordance with autopsy and echocardiography studies showing an increase in LVM to be strongly related to epicardial adipose mass, irrespective of pathological cardiac conditions such as ischaemia and even hypertrophy [8, 21]. Importantly, EAT rather than BMI is more closely related to LVM, which is in line with the findings of Iacobellis and colleagues [8]. Although BMI is considered an important cardiovascular risk factor, the prediction of visceral tissue distribution by BMI, which is an indicator of total adiposity, is governed by the contribution of subcutaneous adipose mass and is therefore flawed [22]. Indeed, visceral adipose tissue (VAT) burden or abdominal adipose tissue is thought to possess a greater risk for the development of diabetes and CAD than BMI [23, 24]. This may be attributable to its distinct functional and anatomical features, namely VAT is more metabolically active and is characterized by a prominent vasculature. Apart from its contiguity to the myocardium and coronary arteries, EAT is in contrast to other visceral fat depots not separated from the myocardium by fascia resembling structures, allowing local paracrine interactions between EAT and the underlying myocardium, rendering EAT most likely a stronger correlate to LVM than general measures of adiposity such as BMI [6]. The mechanism by which EAT influences LV remodelling has not been unravelled yet, but a mechanical and biochemical pathway have been postulated [25]. First, EAT has been shown to reflect central obesity and increased VAT volumes might therefore possess a greater afterload to the left ventricle that comes along with an increased LV output and stroke volume to enable adequate perfusion, prompting cardiac remodelling. Alternatively, there is mounting evidence that EAT is a metabolically active organ and an important source of pro- and anti-inflammatory mediators and cytokines [26]. Arguably, in response to injurious stimuli, the balance shifts from an anti-inflammatory state towards the production and secretion of detrimental adipocytokines such as tumour necrosis factor-α, leptin, resistin and interleukin (IL)-6 and IL-17, which are believed to exert local effects on the underlying coronaries and myocardium [27]. Indeed, these inflammatory markers have been implicated in the pathogenesis of LV remodelling [25]. The local effects of EAT on the myocardium are emphasized by a study by Hua et al. who reported an association between LV function and EAT beyond systemic inflammatory markers and serum levels of adipokines, which is in favour of a site-specific rather than an extra-cardiac VAT effect [28]. Of interest, weight loss has been shown to result in a more pronounced reduction of EAT volume compared to total adiposity measures such as BMI and the waist to hip ratio and was significantly related to a concomitant decrease of LVM compared to BMI changes, suggesting a direct mechanical or functional relationship between these two anatomical compartments [29]. Nevertheless, obesity itself is frequently associated with various cardiovascular risk factors and the metabolic syndrome and is known to be associated with LVM [30, 31]. It is therefore important to examine the impact of EAT on LVM in subjects divided according to their obesity status. Although EAT volume was significantly higher in patients with a BMI > 25, EAT was only associated with LVM in nonobese subjects, after adjustment for BMI and clinical cardiovascular risk factors. These findings highlight the importance of EAT volume in the pathogenesis of LV remodelling. Notably, this phenomenon has also been reported by others, whereby EAT volume was related to incident CAD in only the nonobese patients [32, 33]. Interestingly, the study by Iwayama et al. also found significant differences in adiponectin levels in nonobese subjects according to their CAD status, while in obese patients adiponectin was similar between patients with and without CAD [32]. Similarly, EAT appears only to be associated with the metabolic syndrome and coronary atherosclerosis in nonobese patients [33]. These results highlight the importance of EAT as a marker for cardiovascular risk. Indeed, LVM is strongly associated with adverse outcome and the current results demonstrate that EAT might mediate LV remodelling in otherwise healthy subjects beyond that predicted by BMI. It is plausible that EAT might resolve the so-called obesity paradox, the phenomenon that obesity, as defined by BMI, does not reflect the same burden of risk and that not all obese individuals are similarly exposed to the same future cardiovascular risk [34, 35]. For instance, several studies reported that obesity appeared to be protective against adverse outcome and associated with increased survival and reduced mortality [35]. Interestingly, studies that used surrogate markers for central adiposity such as the waist to hip ratio have depicted a different picture [35]. An increase in central obesity was indeed predictive of worse outcome irrespective of BMI. Presumably, visceral adiposity is more closely related to future cardiac events than BMI, which is not reflective of VAT distribution and is strongly governed by subcutaneous adipose tissue. Our present findings indicate that EAT may explain some of the effect of obesity on LVM in patients with cardiovascular risk factors.

In the absence of flow-limiting epicardial disease, abnormal myocardial perfusion is indicative of coronary microvascular dysfunction, which is considered the functional counterpart of coronary risk factors [36]. The exclusion of haemodynamically significant CAD in the present population allows for the assessment of the coronary microvascular compartment. The coronary microvasculature is often affected by risk factors in an equal manner, thus resulting in a homogeneously increased microvascular resistance. Indeed, a homogeneous perfusion pattern was observed in the present study population. Therefore, quantitative PET imaging is the sole imaging modality to detect this disease entity. The CFR is commonly used to assess coronary vasodilator capacity, which serves as a surrogate marker for microvascular function [19]. However, CFR is the index of maximal achievable MBF relative to resting perfusion and is therefore highly dependent on baseline perfusion, which is dictated by metabolic demands. Moreover, hyperaemic perfusion in turn is governed by heart rate and coronary driving pressure, rendering CFR a parameter that is highly affected by haemodynamic conditions [19]. As such, CMVR is considered a more reliable and quantitative measure of coronary microvascular function [19]. Indeed, the present study showed low predictive ability of the models that employed CFR as a marker for microvascular function. Prior studies suggest EAT to play a key role in the early development of endothelial dysfunction [37–40]. It has been proposed that secreted vasoactive substances by the cardiac fat depot may influence coronary vasomotor function. This hypothesis is strengthened by a recently published study by Bucci et al. who differentiated between intrapericardial and extrapericardial fat and demonstrated that hyperaemic MBF as assessed by quantitative [^15^O]H_2_O PET was only influenced by the intrapericardial fat depot [41]. Mechanisms linking EAT to coronary microvascular dysfunction include changes in the secretion of adipokines, which have been related to insulin resistance and a state of metabolic stress [37]. A decrease in the cardioprotective adiponectin level reflects a pro-atherogenic endothelial milieu with the induction of an inflammatory response resulting in the release of pro-inflammatory and pro-atherogenic cytokines. Indeed, reduced adiponectin levels have been associated with impaired CFR in patients with normal coronary arteries [39]. Similarly, a study by Tok and colleagues showed EAT to predict abnormal coronary Doppler flow measurements in patients with metabolic syndrome [10]. A recent study by Alam et al. among 137 patients without obstructive CAD by CCTA showed EAT to be associated with impaired CFR as determined by ^82^Rb PET even after adjustment for traditional risk factors and coronary calcifications [9]. However, the current study found a moderate correlation between EAT volume and CMVR. After adjustment for traditional risk factors, only LVM was predictive of an increased CMVR in the obese subjects. In accordance with our findings, Brinkley et al. found EAT not predictive of absolute MBF as determined by MRI in asymptomatic subjects [42]. Notably, the myocardium and epicardial fat share the same vasculature; therefore, the presence of myocardial ischaemia may provoke hypoxia of cardiac fat depot, prompting an inflammatory reaction within the EAT resulting in an unfavourable anti-inflammatory/pro-inflammatory metabolic condition with the subsequent secretion of vasoconstrictive and pro-inflammatory cytokines [41]. This may partially explain the discrepancy between EAT volume and absolute myocardial perfusion in subjects without ischaemia. Noteworthy, an interesting finding by the study of Alam et al. is the fact that EAT thickness showed a better correlation with CFR than its volume measurements [9]. Arguably, large portions of the EAT are located distally from vasculature or myocardium. This is illustrated by the finding that periatrial fat, but not periventricular fat, was associated with markers for endothelial dysfunction in patients with atrial fibrillation [43]. Measuring the thickness of EAT intrinsically corrects for these distal portions of the visceral fat surrounding the heart and is probably a better reflection of the vasocrine actions of this fat depot. However, measurements of EAT at a single point are highly dependent on cardiac anatomy and may fail to reflect the total burden of EAT. All in all, data linking EAT to coronary microvascular function provide conflicting results. It is worthy to note that the prevalence of LVM may have attenuated the relationship between EAT and CMVR. A given increase in LVM, albeit not hypertrophy, reduces the capillary density yielding a relative hypoperfusion of the myocardium [44]. As such, the contentious association between EAT and its impact on coronary microvasculature may be justified by the relation between EAT and LVM. Earlier studies examining the impact of EAT on coronary vasculature have not accounted for the impact of LVM. Furthermore, prior studies have only excluded obstructive epicardial disease by means of CCTA, which may have resulted in the inclusion of patients with myocardial ischaemia.

### Study limitations

Other than the limitations inherent to the retrospective nature of the study, some limitations must be acknowledged. First, only EAT has been examined and therefore systemic effects of other visceral fat depots could not be fully excluded. Although the relation between EAT and other visceral fat depots was demonstrated previously, distinct visceral fat origins may exert different effects on the myocardium and coronary microvasculature. Second, data on the prevalence of the metabolic syndrome among study participants were not available and might have provided a better understanding of the relationship between EAT, LVM and CMVR. Third, it is likely that the volumetric amount of EAT does not fully reflect the biochemical properties of this visceral fat depot. Therefore, the assessment of EAT characteristics such as cytokine production, inflammation and/or perfusion might have provided valuable insights. Finally, the applied cut-off value for obesity (BMI ≥ 25) is arbitrary. However, the included patients were free from obstructive CAD and represent therefore a low-risk population. As such, there were only 35 individuals with a BMI > 30. The use of this threshold would have resulted in overfitting of the regression models

### Conclusion

EAT volume is associated with LVM independently of BMI and might therefore be a better predictor of cardiovascular risk than BMI. However, EAT provides no incremental information on coronary microvascular function beyond traditional risk factors and LVM. An increased CMVR is only associated with LVM in the obese subjects.

## Electronic supplementary material

ESM 1 (DOCX 63 kb)
