# Supplementary material for: The impact of obesity on the relationship between epicardial adipose tissue, left ventricular mass and coronary microvascular function
Source: Eur J Nucl Med Mol Imaging. 2015 Jun 9;42(10):1562–73. doi: 10.1007/s00259-015-3087-5 (PMC4521095; doi:10.1007/s00259-015-3087-5)
Supplement: Supplementary file 1 — (DOCX 63 kb) [file 259_2015_3087_MOESM1_ESM.docx]

**Supplementary material**

**Supplementary table 1**

**Male patients (R^2^ = 0.20)**

|  | **Univariable analysis** | | | **Multivariable analysis** | | |
| --- | --- | --- | --- | --- | --- | --- |
|  | **β** | **95% CI** | ***P*-value** | **β** | **95% CI** | ***P*-value** |
| Age | 0.12 | -0.43 – 0.68 | 0.66 |  |  | Ns |
| BMI | 2.53 | 1.10 – 3.67 | < 0.001 | 1.79 | 0.46 – 3.11 | < 0.01 |
| Diabetes | 11.0 | -1.91 – 23.99 | 0.09 |  |  | Ns |
| Hypertension | 4.13 | -6.37 – 14.6 | 0.44 |  |  | Ns |
| Hypercholesterolemia | 0.11 | -11.0 – 11.2 | 0.99 |  |  | Ns |
| Smoking | 5.31 | -4.99 – 15.6 | 0.31 |  |  | Ns |
| Family history of CAD | 4.82 | -5.34 – 15.0 | 0.35 |  |  | Ns |
| EAT | 0.18 | 0.09 – 0.27 | < 0.001 | 0.11 | 0.01 – 0.22 | 0.04 |
| CAC score | 0.01 | -0.02 – 0.01 | 0.35 |  |  | Ns |

**Female patients (R^2^ = 0.22)**

|  | **Univariable analysis** | | | **Multivariable analysis** | | |
| --- | --- | --- | --- | --- | --- | --- |
|  | **β** | **95% CI** | ***P*-value** | **β** | **95% CI** | ***P*-value** |
| Age | 0.19 | -0.56 – 0.18 | 0.31 |  |  | Ns |
| BMI | 1.84 | 1.03 – 2.65 | < 0.001 | 1.23 | 0.33 – 2.13 | < 0.01 |
| Diabetes | 5.61 | -4.89 – 16.1 | 0.29 |  |  | Ns |
| Hypertension | 5.11 | -2.58 – 12.8 | 0.19 |  |  | Ns |
| Hypercholesterolemia | 1.18 | -6.78 – 9.13 | 0.77 |  |  | Ns |
| Smoking | 6.96 | -0.21 – 14.1 | 0.06 | 6.82 | 0.31 – 13.3 | 0.04 |
| Family history of CAD | 0.55 | -6.86 – 7.95 | 0.88 |  |  | Ns |
| EAT | 0.16 | 0.08 – 0.24 | < 0.001 | 0.13 | 0.04 – 0.22 | < 0.01 |
| CAC score | 0.00 | -0.03 – 0.03 | 0.87 |  |  | Ns |

CI, confidence interval; BMI, body mass index; CAC, coronary artery calcium score; CAD, coronary artery disease; EAT, epicardial adipose tissue; NS, not significant.
